# Prevalence and Mode of Birth in Late Fetal Mortality in Spain, 2016–2019

**DOI:** 10.3390/ijerph20031777

**Published:** 2023-01-18

**Authors:** Pedro Hidalgo-Lopezosa, Ana María Cubero-Luna, Rubén García-Fernández, Andrea Jiménez-Ruz, María Isabel Maestre-Luna, Cristina Liébana-Presa, María Aurora Rodríguez-Borrego, Pablo Jesús López-Soto

**Affiliations:** 1 Instituto Maimónides de Investigación Biomédica de Córdoba (IMIBIC), 14004 Córdoba, Spain; 2 Departamento de Enfermería, Farmacología y Fisioterapia, Universidad de Córdoba, 14004 Córdoba, Spain; 3 SALBIS Research Group, Faculty of Health Sciences, Campus de Ponferrada, Universidad de León, 24401 León, Spain; 4 Hospital Universitario Reina Sofía, 14004 Córdoba, Spain

**Keywords:** stillbirth, late fetal mortality, mode of birth, cesarean birth

## Abstract

(1) Background: The rate of cesarean sections in late fetal mortality remains high. We aimed to determine the prevalence of late fetal mortality in Spain and risk factors for cesarean birth in women with stillbirth ≥ 28 weeks gestation between 2016–2019. (2) Methods: A retrospective observational study with national data between 2016–2019. A total of 3504 births with fetal dead were included. Sociodemographic, obstetrical and neonatal variables were analyzed using univariate and multivariate logistic regression (MLR), with cesarean birth with a stillborn ≥ 28 weeks gestation as the dependent variable. (3) Results: The late fetal mortality rate was 2.8 × 1000; 22.7% of births were by cesarean section. Factors associated with cesarean were having a multiple birth (aOR 6.78); stillbirth weight (aOR 2.41); birth taking place in towns with over 50,000 inhabitants (aOR 1.34); and mother’s age ≥ 35 (aOR 1.23). (4) Conclusions: The late fetal mortality rate increased during the period. The performance of cesarean sections was associated with the mother’s age, obstetric factors and place of birth. Our findings encourage reflection on how to best put into practice national clinical and socio-educational prevention strategies, as well as the approved protocols on how childbirth should be correctly conducted.

## 1. Introduction

The global world rate of fetal deaths from 22 weeks of gestation (wg) is estimated at 18.4 per 1000 births; this rate is equivalent to about 2.6 million fetal deaths each year [1]. In addition, it is estimated that around one or two million deaths cannot be assessed due to the difficulty of measuring their prevalence, especially in those countries with limited access to health care [2].

Late fetal death is defined as a “death occurring at or after 28 wg, before the complete expulsion or removal of the product of conception from the mother’s body, regardless of the duration of gestation” [3]. Perinatal mortality is estimated by adding together fetal deaths and early neonatal mortality, which are deaths that occur in the first seven days after birth [4]. Regarding stillbirth, however, there has been relatively little research into this area and, globally, it is not usually included in many major maternal and child health strategies [5]. There are also differences in the way some countries report the gestational age and birth weight criteria linked to stillbirths, which further hinders international comparisons. Due to these differences, the World Health Organization has recommended limiting international comparisons to third trimester stillbirths, which are defined by a birth weight limit of 1000 g or a gestational age limit of 28 full weeks [6]. The gestational age limit of 28 full weeks is often used because the criteria for stillbirth registration in most countries are based on gestational age [7].

A total of 97% of late fetal deaths occur in developing countries, which have a prevalence of 3% of all births. In developed countries, the prevalence is below 1% [2]: for instance, while the stillbirth rate in sub-Saharan Africa is as high as 32.2 × 1000 births [8], in Europe, most countries have late fetal mortality rates of 2.0–3.5 × 1000 births. Stillbirth rates fell between 2010 and 2015, but the trends were highly variable. Some countries, such as the Netherlands, Poland, Scotland and England and Wales, saw significant reductions in their late fetal death rates, while, in other countries, the rates remained stable or increased: in 2015, the highest rates of late fetal death were found in Bulgaria and Romania, with 5.7 and 3.6 × 1000, respectively. Other countries in Europe also showed high figures, such as France with 4.8 × 1000, 3.9 × 1000 for Luxembourg or 3.7 × 1000 for Hungary. The lowest were recorded in Cyprus with 1.4 × 1000, and in Denmark and Iceland with 2 × 1000 [7]. Spain is in an average position with 2.7 × 1000, and actually recorded a 0.1 increase compared with the 2015 figures [9]. Further research should be carried out by health authorities into the changes in stillbirth rates in 2010 and 2015 [7].

The etiology of fetal death is often unknown, despite being the subject of many studies [7,10]. In many cases, data on fetal deaths are not collected or their causes are not specified, producing a gap in our knowledge that hinders the creation of prevention strategies [11]. In fact, when it comes to high-income countries, the proportion of late fetal deaths of unknown cause is high, and could be considerably improved if data collection and increased research were encouraged in this field [12]. In fact, a recent study by the International Stillbirth Alliance estimates that high-income countries, such as Spain, could reduce their fetal mortality rate by up to a third by implementing clinical and socio-educational prevention strategies [12].

Recommendations on the management and birth mode in the case of dead fetuses state that induced labor and the vaginal route should be given priority because they produce fewer complications, and because the woman can be discharged from hospital earlier [13]. In fact, this mode is usually tried as there are no reasons to accelerate birth, so improving the child’s wellbeing cannot be cited as a reason for conducting a cesarean section; although, to help the mother, it may be necessary if complications such as hemorrhages, uterine discharge, pre-eclampsia, placental abruption or other serious difficulties occur, which require immediate evacuation of the uterus [13]. Several factors can also influence the birth mode in women with a dead fetus. In some studies, it has been linked to the volume of births in the hospital, teaching programs for obstetrics residents or other factors, such as advanced gestational age, parity and black race [14]. Other authors link the birth mode to onset, spontaneous or induced, and to whether the mother has a medical history of cesarean sections [15]. Abdominal birth involves greater risks for the mother. About 23% of women who had a cesarean section with a dead fetus presented complications arising from the operation, mainly because it is a major factor to take into account for future births [16].

The aim of this study was to determine the prevalence of late fetal mortality and risk factors for cesarean birth in women with a dead fetus of 28 or more weeks of gestation in Spain from 2016 to 2019 (period corresponding to the latest official data).

## 2. Materials and Methods

### 2.1. Study Design and Population

This observational and analytical retrospective study employed the official information sources given by the Spanish National Institute of Statistics (INE) [17] on late fetal mortality. We included data of births resulting in a stillborn in gestations lasting for 28 weeks or more, between January 2016 and December 2019.

### 2.2. Sample and Inclusion Criteria

A total of 3504 births were included, all with a gestational age of 28 weeks or more and which produced a stillborn at birth. Births under 28 wg were excluded. It is important to point out that it was the parents or relatives who registered the variables, which accounts for missing data in some of the variables; for example, the number of weeks of gestation. In fact, it is estimated that between 2010 and 2015 the underreporting rate in Spain for stillbirths of more than 28 wg was 7.6% [18,19].

### 2.3. Study Variables

The variables studied were: (i) maternal age (≥19/20–29/30–34/≥35 years); mother’s province of residence (Spanish province); educational level (primary or lower/secondary/university); country of origin (Spanish/rest of Europe/Africa/America/Asia-Oceania); mother’s marital status (married/single/separated-divorced); mother’s occupation (active/unemployed); place of birth (hospital/home/others); size of town/city of residence (up to 10,000/10,001–20,000/20,001–50,000/50,001–100,000/>100,000); number of fetuses (single/multiple); parity (primiparous/multiparous); gestational age (28–36/37–42/>42 wg); term birth (yes/no); type of birth (vaginal/cesarean section); intergenic interval (months); stillborn sex (male/female) and stillborn weight (grams).

### 2.4. Data Analysis

For the data analysis, we caried out a descriptive analysis of the variables, with the categorical and ordinal variables expressed as numbers (*n*) and percentages (%), while the numerical variables were expressed as mean and standard deviation (SD). Then, the risk of the independent variables for the cesarean birth variable and the crude odds ratio (OR) were calculated. Finally, a multiple logistic regression analysis (MLR) was performed with the independent variables which obtained the most significant values in the univariate analysis. Using the Wald statistic, we individually removed variables with a *p* value ≥ 0.15 from the model, and studied the possible interactions between the variables. Variables with a significance of over 0.05 were considered possible confounding factors. The goodness of fit of the model was calculated using the Hosmer-Lemeshow statistic, with a value of *p* < 0.05 considered significant. To carry out the statistical analysis, we used the following program: IBM SPSS Statistics version 25.

### 2.5. Ethical Considerations

Data for the study were available and freely accessible on the INE website. Therefore, when using secondary data, no report from the ethics committee was required in accordance with Spanish legislation. Informed consent was not required and the study complied with the Helsinki Guidelines.

## 3. Results

For the period studied, according to the National Institute of Statistics, the number of late fetal deaths was 4141 total dead fetuses, which is equivalent to a rate of 2.7 × 1000. Taking into account the rate of underestimation of cases (around 7%) [18,19], if a percentage increase of 5% were applied, a conservative estimate of the rate of late fetal death in Spain for that time period would be 2.8 × 1000.

We analyzed a total of 3504 births resulting in fetal death, of which 9.8% were multiple births. A total of 2740 (94.3%) of fetal death occurred prior to delivery, 166 occurred during delivery (5.7%) and 598 were missing data. As can be seen in Table 1, the mean age of the women was 32.9 (±5.9) years (minimum 14 years and maximum 54 years); 2.5% were under 20 years old and 42.8% were 35 or older. Most of the cases were in the provinces of Barcelona (14.4% of the total), Madrid (11.9%), Valencia (5.4%), Alicante (4.8%) and Seville (4%). The provinces with the lowest percentage of cases were Cuenca, Guadalajara, Huesca, Soria, Segovia and Lugo, with a figure of 0.1%; 99.6% of the births took place in hospital.

The mean number of weeks of gestation after which the birth occurred was 34.7 (3.8) weeks, with a minimum of 28 and a maximum of 42. Furthermore, 37.2% were term births and 62.8% were preterm; 77.3% were vaginal births, while the remaining 22.7% required a cesarean section, including women with a medical history of previous cesarean sections. An total of 52.8% were primiparous, 40% were married mothers and 60% were single, separated or divorced. Only 20% had studied at university level. With regards to country of origin, 75.8% were Spanish, 5.4% from the rest of Europe, 11.9% from Africa, 4.7% from America and 2.2% from Asia and Oceania. The global rate of cesarean births in towns/cities with over 50,000 inhabitants was 23.4%, while the figure for towns/cities with under 50,000 inhabitants was 18.3%. Table 1 shows all data.

Table 2 shows the differences in the percentages of vaginal births and cesarean sections in women with a dead fetus according to parity. In primiparous women, statistically significant differences were found by age: women aged 35 or over had 27.3% cesarean sections, while those under 35 years had 19.6% (*p* < 0.001). Among multiparous women, statistically significant differences were found when comparing this data between births in towns/cities of 50,000 inhabitants or more (24% cesarean sections) with smaller towns/cities (17.8% cesarean sections; *p* = 0.044); women of Spanish nationality had a higher rate of cesarean sections than foreign women (24.6% versus 19.2%, *p* = 0.032); this also occurred in women with fetuses of 4000 g or over compared to lower weights (38.9% versus 22.1%; *p* = 0.018). In both groups, the cesarean section rate was higher than that recorded in women with multiple births.

Table 3 shows the findings from the RLM model. Factors associated with cesarean section in women with stillborn after 28 weeks were: having a multiple or twin birth (OR 6.78, 95% CI 5.22–8.80; *p* < 0.001); stillborn weight (OR 2.41, CI 95% 1.46–3.97; *p* = 0.001); births that took place in towns/cities with over 50,000 inhabitants (OR 1.34, CI 95% 1.01–1.78, *p* = 0.041); and mother’s age of 35 or over (OR 1.23; 95% CI 1.03–1.48; *p* = 0.022).

## 4. Discussion

According to the latest report from the European Perinatal Health Report (EUROPERISTAT), published in 2015, when compared with the data from the previous 2010 report, the rate of late fetal deaths has decreased in some European countries, such as Cyprus, Belgium, Denmark, Ireland, Greece, Malta, Holland, Poland, Romania, Lithuania, Austria, England and Norway. However, there are also many countries where these deaths have increased, as in Germany, Estonia, Spain, France, Luxembourg, Hungary, Portugal, Slovenia, Slovakia, Finland, Sweden, Iceland and Switzerland [7]. This data calls for a reflection from the scientific community, because, rather than decreasing (as one would expect), in Spain and in many neighboring countries, the figures seem to be on the rise. Although it is true that perinatal mortality is falling, all the signs point to this fall occurring at the expense of early neonatal mortality. Greater efforts are therefore needed to determine the causes of late fetal death [5]. In most cases, the cause of fetal death remains unknown, even after performing the autopsy [13].

In the present study, although analysis does not focus on the causes of late fetal mortality, prematurity occupies 2/3 of the sample, with great prematurity (<32 weeks) accounting for 31.7%. Note that prematurity is known to be associated with the risk of late fetal death [9], and it is considered as a number 10 group according to the Robson 10-step classification, a clinically relevant and useful classification system to improve outcomes [20].

The mode of birth after fetal death depends on the gestational age, the mother’s obstetric history and even her preferences, so it is up to health professionals to assess the risks and benefits in each case in order to achieve the most favorable results. The most common option consists of inducing labor with misoprostol to attempt vaginal birth and thus avoid cesarean section [21]; however, this operation presents greater complications and affects the woman’s future fertility [22]. In the present work, we presented data on the final birth route rather than on induction rates. Our findings show that 22.7% of the women had a cesarean section, with very similar percentages in primiparous and multiparous women. Note that the total cesarean section rate in Spain for 2015 was 24.6% [7], and that it has increased in the last few years [23]. Similarly, other studies found percentages of 21% [16] or around 27% [24], although most found lower percentages of around 10% [25]. Generally, however, most studies have reported lower cesarean rates than in live births [22]. According to one of these studies, around 40% of first cesarean sections with dead fetuses had not received clear medical advice recommending a cesarean section [15].

Our findings show that the factors associated with cesarean birth were the mother’s advanced age, multiple births, fetus weight and the size of the town/city where the birth took place. Ramseyer et al. also found that older women had a higher risk of cesarean section [14]. Other authors did not find this link, although they associated other factors, such as a medical history of previous cesarean sections [24] or the increase in gestational age, with a higher ratio of third-trimester cesarean sections [15,24], which was not noted in the present work. Another risk factor for cesarean section with a stillborn in this study was multiple births, which is logical since it is also a risk factor for cesarean section in live fetuses [26].

Regarding the fetus weight, coinciding with our results, Rossi et al. found that the risk of cesarean section increased in large for gestational age fetuses [24]. One notable statistic was the higher risk of cesarean section when the birth took place in bigger towns/cities. To our knowledge, no similar results have been found which assess this variable in the case of dead fetuses, although some studies have related a generally higher risk of cesarean section in urban populations [27].

The main limitation of this study lies in the rate of under-reporting of data to the National Institute of Statistics (INE) by parents or relatives, which accounts for the missing data in some variables. In fact, the data declared by the INE on late fetal deaths do not appear to tally with those shown in this study, although it must be remembered that our results have been obtained from birth records, while the data issued by the INE include the total number of dead fetuses (including those from multiple births). We believe that the INE has taken into account other variables in addition to gestational age to determine the status of late fetal death, or that there must be considerable variation in the limit of 28 wg included in the family’s declaration of this data. However, regardless of the under-reporting of the data, the number of records included 3504 births, and the fact that the total number of late fetal deaths during a 4-year interval were used allows us to generalize the results, which is the main strength of our study. Other limitations were the missing data on causes of cesarean sections and those derived from retrospective studies. In addition, we have a crude cesarean section rate, which is not very objective data by which to identify whether cesarean sections were indicated following clinical recommendations in the guidelines or for unjustified reasons.

## 5. Conclusions

In summary, late fetal mortality in Spain still shows an upward trend. In addition, the route of birth chosen in late fetal death can affect future births. The percentage of cesarean sections was high and could be associated with multiple or twin births, larger fetuses, the birth taking place in towns/cities with over 50,000 inhabitants and mothers over 35 years of age. Our findings emphasize the need to promote research into the causes of late fetal mortality, as well as to implement clinical prevention strategies in the birth process. Health professionals should put into practice the official national protocols on managing births with a dead fetus in order to reduce the number of cesarean sections, limiting their use to specific cases.

## Figures and Tables

**Table 1:** Sociodemographic, obstetric and fetal characteristics in the national cohort studied, 2016–2019 (*n* = 3504).

Variable	*n*	(%)
Mother’s age (years) mean (SD)	32.9 (±5.9)	
≤19	88	(2.5)
20–29	822	(23.5)
30–34	1096	(31.3)
≥35	1498	(42.8)
Level of education		
Primary school or below	423	(15.4)
Secondary school	1771	(64.5)
University	549	(20)
Country of origin		
Spain	2639	(75.8)
Rest of Europe	188	(5.4)
Africa	414	(11.9)
America	163	(4.7)
Asia/Oceania	76	(2.2)
Marital status		
Married	1401	(40.1)
Single	1995	(57)
Separated/divorced	100	(2.9)
Parity		
Primiparous	1851	(52.8)
Multiparous	1653	(47.2)
Multiple birth	343	(9.8)
Weeks of gestation mean (SD)	34.7 (±3.8)	
28–32	1111	(31.7)
32–36	1090	(31.1)
37–42	1303	(37.2)
Birth mode		
Vaginal	2709	(77.3)
Cesarean	795	(22.7)
Sex of NB		
Male	1842	(52.6)
Female	1662	(47.4)
Fetus Weight (grs) mean (SD)	2222 (±918)	
<2500	1770	(50.5)
2500–3500	969	(27.7)
≥4000	765	(21.8)
Complications during birth		
Yes	742	(21.2)
No	2762	(78.8)
Year of birth		
2016	948	(27.1)
2017	906	(25.9)
2018	835	(23.8)
2019	815	(23.3)
Interpregnancy interval (months) mean (SD)	32.7 ± 44	

**Table 2:** Birth mode according to parity in women with a dead fetus from 28 wg. *n* = 3504.

Variable	Primiparous			Multiparous		
	Vaginal *n* = 1620 *n* (%)	Cesarean *n* = 468 *n* (%)	*p*-Value	Vaginal *n* = 1089 *n* (%)	Cesarean *n* = 327 *n* (%)	*p*-Value
Mother’s age						
<35 years	1061 (80.4)	258 (19.6)		534 (77.7)	153 (22.3)	
≥35 years	559 (72.7)	210 (27.3)	<0.001	555 (76.1)	174 (23.9)	0.476
Marital status						
Married/with partner	504 (75)	168 (25)		548 (75.2)	181 (24.8)	
No partner	1114 (78.8)	299 (21.2)	0.049	537 (78.7)	145 (21.3)	0.112
Level of education						
Up to secondary	910 (77.2)	268 (22.8)		777 (76.5)	239 (23.5)	
University	264 (74.4)	91 (25.6)	0.261	150 (77.3)	44 (22.7)	0.799
Town/city of birth						
<50 K inhabitants	218 (81.3)	50 (18.7)		180 (82.2)	39 (17.8)	
≥50 K inhabitants	1402 (77)	418 (23)	0.114	909 (75.9)	288 (24.1)	0.044
Country of origin						
Spanish	1253 (77.2)	371 (22.8)		765 (75.4)	250 (24.6)	
Other	355 (78.7)	96 (21.3)	0.483	315 (80.8)	75 (19.2)	0.032
Gestational age						
Term	587 (77.9)	167 (22.1)		420 (76.5)	129 (23.5)	
Premature	1033 (77.4)	301 (22.6)	0.827	669 (77.2)	198 (22.8)	0.774
Multiple birth						
Yes	78 (37.1)	132 (62.9)		57 (42.9)	76 (57.1)	
No	1542 (82.1)	336 (17.9)	<0.001	1032 (80.4)	251 (19.6)	<0.001
Fetus weight (g)						
<4000	1364 (78.1)	382 (21.9)		919 (77.9)	261 (22.1)	
≥4000	24 (68.6)	11 (31.4)	0.177	22 (61.1)	14 (38.9)	0.018

X^2^ test was used for the comparison of categorical variables.

**Table 3:** Factors associated with cesarean birth in stillbirths of over 28 wg.

Variable	Analysis	
	Univariant	Multivariant
	*p*, Crude OR CI (95%)	*p*, Adjusted OR CI (95%)
Age (years)		
<35	Reference	
≥35	<0.001, 1.33 (1.14–1.57)	0.001, 1.23 (1.03–1.48)
Marital status		
Married/partner	Reference	
Separated/no partner	0.010, 0.81 (0.69–0.95)	
Country of origin		
Spanish	0.054, 1.20 (0.99–1.45)	
Other	Reference	
Multiple birth	<0.001, 6.75 (5.34–8.54)	<0.001, 6.78 (5.22–8.80)
Size of town/city of birth		
<50,000 inhabitants	Reference	
≥50,000 inhabitants	0.012, 1.36 (1.07–1.74)	0.041, 1.34 (1.01–1.78)
Parity		
Primiparous	Reference	
Multiparous	0.637, 1.04 (0.88–1.22)	
Intergenesic interval < 24 m	0.004, 1.26 (1.07–1.47)	
Gestation age (weeks)	0.227, 1.01 (0.99–1.03)	
Fetus Weight (g)	0.001, 1.93 (1.17–3.16)	0.001, 2.41 (1.46–3.97)
Complications at birth	<0.001, 12.36 (9.9–15.5)	

Multiple Logistic Regression analysis: R^2^: 0.113; Hosmer-Lemeshow: 0.956.

## Data Availability

The data presented in this study are available on request from the corresponding author.
